# Using Canadian administrative health data to examine the health of caregivers of children with and without health problems: A demonstration of feasibility.

**DOI:** 10.23889/ijpds.v4i1.584

**Published:** 2019-04-02

**Authors:** Jamie C Brehaut, Anne Guèvremont, Rubab G Arim, Rochelle E Garner, Anton R Miller, Kimberlyn M McGrail, Marni Brownell, Lucyna M Lach, Peter L Rosenbaum, Dafna E Kohen

**Affiliations:** 1 Ottawa Hospital Research Institute, Clinical Epidemiology Program, The Ottawa Hospital, General Campus, 501 Smyth Road, Room 1284 Centre for Practice-Changing Research; Box 201B, Ottawa, Ontario, K1H 8L6, Canada; 2 Health Analysis Division, Statistics Canada, 100 Tunney’s Pasture Driveway, Ottawa, Ontario, K1A 0T6, Canada; 3 Department of Pediatrics, Faculty of Medicine, University of British Columbia, 4480 Oak Street, Vancouver, British Columbia, V6H 3V4, Canada; 4 School of Population and Public Health, Faculty of Medicine, University of British Columbia, 2206 East Mall, Vancouver, British Columbia, V6T 1Z9, Canada; 5 Department of Community Health Sciences, Max Rady College of Medicine, Rady Faculty of Health Sciences, University of Manitoba, 727 McDermot Avenue, Winnipeg, Manitoba, R3E 3P5, Canada; 6 School of Social Work, McGill University, 3506 University Street, Montreal, Quebec, H3A 2A7, Canada; 7 Department of Paediatrics, Faculty of Health Sciences, McMaster University, 1280 Main Street West, Hamilton, Ontario, L8S 4L8, Canada; 8 Centre for Research in Evidence-Based Practice, Bond University, Gold Coast, QLD, Australia

**Keywords:** Population data, linked data, case-mix, children with special health care needs

## Abstract

**Introduction:**

Caregivers of children with health problems experience poorer health than the caregivers of healthy children. To date, population-based studies on this issue have primarily used survey data.

**Objectives:**

We demonstrate that administrative health data may be used to study these issues, and explore how non-categorical indicators of child health in administrative data can enable population-level study of caregiver health.

**Methods:**

Dyads from Population Data British Columbia (BC) databases, encompassing nearly all mothers in BC with children aged 6-10 years in 2006, were grouped using a non-categorical definition based on diagnoses and service use. Regression models examined whether four maternal health outcomes varied according to indicators of child health.

**Results:**

162,847 mother-child dyads were grouped according to the following indicators: Child High Service Use (18%) vs. Not (82%), Diagnosis of Major and/or Chronic Condition (12%) vs. Not (88%), and Both High Service Use and Diagnosis (5%) vs. Neither (75%). For all maternal health and service use outcomes (number of physician visits, chronic condition, mood or anxiety disorder, hospitalization), differences were demonstrated by child health indicators.

**Conclusions:**

Mothers of children with health problems had poorer health themselves, as indicated by administrative data groupings. This work not only demonstrates the research potential of using routinely collected health administrative data to study caregiver and child health, but also the importance of addressing maternal health when treating children with health problems.

## Introduction

Parents of children with health problems experience challenges beyond those experienced by parents of healthy children. These include increased time spent providing care [1, 2]; impacts on family resources such as lower incomes [3, 4], increased material hardship [5, 6]; employment constraints [3-5, 7]; and child care and support challenges [7, 8]. Demographic changes such as smaller family units and more single-parent families [9] may also contribute to greater challenges for parents and families of children with health problems.

While many families cope well, some experience significant psychological and physical health challenges [10]. Caregivers of children with health problems do not only demonstrate poorer psychological health, including greater stress, distress, emotional problems, and depression, compared to caregivers of children without health problems [5, 11, 12], they also show a greater number of chronic physical conditions [5]. Importantly, such effects do not appear to be limited to specific types of child disability, nor are they limited only to the most severe. Instead, use of large-scale survey datasets has shown that a substantial number of families caring for children with health problems are dealing with physical and psychological health issues themselves [13, 14].

As the need for interventions to address the challenges faced by families of children with health problems becomes more apparent [15], so does the need for flexible, population-based health measures. To date, population-based studies have relied on survey data, which have important limitations. Population-based health surveys are resource intensive, expensive to administer, often offer limited coverage of children with specific health problems, lack consistent measures, and are subject to self-reported data which has the limitations of recall bias, attrition over time, and shared method variance biases due to single respondents[16-18]. Administrative health data, on the other hand, may provide relatively economical, consistent, and objective data on children and families across broad populations, yet remains underutilized outside of health services research, especially for pediatric populations.

This study examines how routinely collected provincial administrative health data might be used to compare the health of caregivers of children with and without health problems. Our goals for this work are two-fold. First, we wish to demonstrate that maternal health associations with child health can be measured at a population level when self-reported surveys are not available. Second, we wish to explore whether two previously developed [16, 19] ‘non-categorical’ measures of child health, i.e., measures that identify groups by common challenges and consequences rather than by disease state [20], have associations with maternal outcomes when implemented in administrative data. This work could enable the use of such tools for studying large-scale, policy-motivated questions designed to improve the health and well-being of families of children with health problems.

## Methods

### Sample and data source

The cross-sectional sample for this study included children aged 6 to 10 years and their mothers enrolled in the British Columbia (BC) Medical Service Plan for the year 2006. We chose the year 2006 because these were the most recent data available to us at the time of the study, given that the cohort was part of a larger longitudinal study that focused on the health trajectories of caregivers, and included a seven-year follow-up period [21].

We limited the sample to school-age children because many childhood health conditions are not diagnosed until school-age and to avoid the inclusion of short term perinatal maternal health issues in our maternal health outcomes. Children were linked to mothers based on the provincial Medical Service Plan (MSP) contract number, which is the same for all family members. Only children linked to one mother were included (88% of cases; children could be linked to more than one mother in cases of marital separation and family dissolution).

This study uses linked data from five separate administrative data holdings of Population Data BC (PopData) to identify children with health problems and maternal outcomes:

1. The *Medical Services Plan (MSP) Payment Information File*:[22] medical services provided by fee-for-service practitioners to people covered by BC’s universal health insurance program from 1985 to the present.
2. The *Discharge Abstract Database (DAD)*:[23]hospital discharges, transfers, and in-hospital deaths of patients from all acute-care hospitals from 1985 to the present.
3. *PharmaNet*:[24] prescription data for drugs and medical supplies (for example, insulin pumps, orthotics) dispensed by pharmacies.
4. The *Consolidation File* (MSP Registration and Premium Billing):[25] population demographic data prepared for research use by PopData.
5. *Aggregated Diagnosis Groups (ADG) data*: generated by PopData using the Johns Hopkins Adjusted Clinical Groups (ACG) Case-Mix System software version 10.0 [26], described below.

All PopData data are linked using encrypted, randomly-assigned identifiers that match personal health numbers across databases and thereby provide individual level data that maintain anonymity and confidentiality.

### Data access

The study was approved by Research Liaison staff of PopData, the Data Stewards at the BC Ministry of Health, and the Ottawa Health Science Network Research Ethics Board, where the research was carried out. The encrypted data files were made accessible to the research team on a secure research environment through PopData.

### Identifying children with health problems: High Service Use and Diagnosis indicators

Our High Service Use indicator identified children with elevated health care service use based on two validated survey instrument indicators, one based on medication use and one on physician visits. This approach was modeled on the first two items of the Children with Special Health Care Needs (CSHCN) Screener tool [19]. To measure medication use, we identified children who had at least 9 months (≥274 days) of medicine use in any 365-day period from January 2005 to December 2007. A day of medicine use was defined based on the date the prescription was filled and the number of days of medication supplied in the PharmaNet data. One day of prescription medication use could include one or more medications prescribed for that day. To measure physician visits, we examined the distribution of the number of children’s physician visits in the MSP data and defined age-specific cut-offs to identify children at or above the 95th percentile. Children who met the criteria for one or both measures were identified as having High Service Use.

Our Diagnosis indicator identified children who had a major and/or chronic condition as defined by the John Hopkins ACG Case-Mix System [27, 28]. The system classifies clinical groupings into one of 32 ADGs based on persistence, stability, and severity. Twelve of these ADGs are considered major (i.e., expected prognosis involves disability or death) and/or chronic (i.e., likely to persist for more than 12 months) for children ages 0-17 [16, 26].

Children with health problems were identified based on each indicator separately (High Service Use vs. Not; Diagnosis vs. Not) and combined (both High Service Use and Diagnosis vs. neither). Previous work explored the utility of using these indicators individually as non-categorical measures of child health, but did not examine them in combination [16, 19] or in association with maternal health outcomes. In order to validate the use of the indicators together, we explored their association with specific child health outcomes (e.g. number of different specialists visited, number of days in hospital). Appendix 1 describes all child outcomes measured.

Some mothers (23% of the sample) were linked to more than one child because they had more than one child aged 6 to 10 in 2006. If mothers were linked to two or more children, their category was selected based on the child identified with the most severe health problem as defined by our four groups, in the following order: 1) Both indicators (most severe), 2) High Service Use indicator, 3) Diagnosis indicator, and 4) Neither (least severe).

### Maternal health outcomes

Maternal health outcomes are described in Appendix 1. Several outcomes were chosen based on survey data studies showing differences according to child health groupings [5, 14]. Related health and service use outcomes were chosen in order to take advantage of the breadth of administrative data available (e.g., lab visits, x-rays etc.). For drug use, we also included number of different level-3 Anatomical Therapeutic Chemical (ATC) codes. Each drug identification number (DIN) has a World Health Organization ATC classification code assigned by Health Canada [29].The ATC structure divides active substances into groups according to the organ or system on which they act and their therapeutic, pharmacological, and chemical properties. The number of different level-3 ATC codes was selected because Level-3 ATC codes represent major therapeutic or pharmacological subgroups. We also included an indicator of the percentage of mothers with any chronic condition and another indicating any mood or anxiety disorder. All chronic conditions were defined based on specific International Classification of Diseases v.9 (ICD-9) diagnostic codes (see Appendix 1 for codes).

### Analyses

Due to the very large sample size in this study, very small differences emerged as statistically significant. We used effect size as a way of comparing the size of differences; Cohen’s d was used for all comparisons using established criteria for small (0.2 to 0.5), medium (0.5 to 0.8), large (0.8 to 1.2), and very large (more than 1.2) effect sizes based on the standardized mean difference [30, 31]. Regression models further explored associations between child health groupings and four maternal health outcomes (number of maternal physician visits, any maternal chronic condition, maternal mood or anxiety disorder, maternal hospitalization), chosen in order to have one physician services-based, one physical, one psychological health measure, and one measure based on hospital use. A linear regression was conducted for the number of maternal physician visits, and logistic regression performed for the other three binary outcomes. All regression models controlled for maternal age, child age and sex, and two family socio-economic indicators, neighbourhood income quintile[32] and receipt of a premium subsidy (BC residents must pay a premium for health services, relief from which can be obtained through a needs-based subsidy).

## Results

All children registered with the BC MSP and aged 6 to 10 in 2006 constituted a sample of 232,670 children. Based on the selection rules outlined above (children linked to only one mother, one child selected per mother), the final sample included 162,847 mother-child dyads.

Table 1 describes characteristics across child health groupings separately (High Service Use (18%) vs. Not (82%); Diagnosis (12%) vs. Not (88%)) and combined (Both High Service Use and Diagnosis (5%) vs. Neither (75%)). These groupings did not differ in terms of age of the mother (mean age of total sample, 38.1 years), age of the child, or sex of the child. The groups were also similar on our two proxy measures of socioeconomic status, namely proportion living in a lowest-income quintile area, and proportion having received a premium subsidy.

**Table 1: Family, maternal, and child characteristics of the sample (N=162,847) across child health groupings:** Note: Effect size for all differences was less than 0.20 (less than a small effect size).

	Child High Service Use		Child Diagnosis		High Service Use and Diagnosis combined	

	Yes	No	Yes	No	Both	Neither

	n = 29,480	n = 133,507	n = 19,892	n = 132,955	n = 8,665	n = 122,280
	(18%)	(82%)	(12%)	(88%)	(5%)	(75%)
**Maternal & family characteristics**						
Age (mean years (sd))	37.93 (5.85)	38.08 (5.87)	38.20 (5.71)	38.03 (5.89)	38.10 (5.73)	38.06 (5.88)
Living in lowest-income quintile area (%)	20.86	19.41	19.72	19.67	20.66	19.45
Receiving premium subsidy (%)	30.58	23.52	26.67	24.53	30.03	23.47
**Child characteristics**						
Age (mean years, (sd))	8.00 (1.45)	8.08 (1.41)	8.03 (1.42)	8.07 (1.41)	8.00 (1.45)	8.08 (1.41)
Male (%)	55.36	50.58	54.31	51.04	55.76	50.34

Table 2 describes a variety of child outcomes across our child groupings. As expected, and consistent with our previous work [16, 19], the Both and High Service Use groups showed large or very large effect sizes when compared to the Neither or Non High Service Use groups on most service-based child outcomes, while somewhat smaller but non-trivial effect sizes were observed for the Diagnosis group compared to the Non Diagnosis group. This pattern held for number of physician visits, laboratory visits, different specialists seen, number and days with a prescription, and days hospitalized. These results show that this approach yields groups of children that differ on the outcomes examined.

**Table 2: Validation of child health groupings:** ^S^small effect size compared to corresponding “no” or “neither” groups ^M^medium effect size compared to corresponding “no” or “neither” groups ^L^large effect size compared to corresponding “no” or “neither” groups ^XL^extra-large effect size compared to corresponding “no” or “neither” groups ^*^These variables were not components of the definitions of Child High Service Use or Child Diagnosis.

	Child High Service Use		Child Diagnosis		High Service Use and Diagnosis combined	

	Yes	No	Yes	No	Both	Neither

	n = 29,480	n = 133,507	n = 19,892	n = 132,955	n = 8,665	n = 122,280
	(18%)	(82%)	(12%)	(88%)	(5%)	(75%)
**Child characteristics (mean (sd))**						
Number of physician visits (excluding lab and x-ray visits)	8.97 (6.00)^XL^	2.51 (2.32)	7.36 (5.76)L	3.16 (3.55)	10.99 (6.76)XL	2.32 (2.22)
Number of lab visits	1.35 (3.08)^L^	0.21 (0.54)	1.20 (3.45)^M^	0.31 (0.83)	2.27 (4.97)^XL^	0.20 (0.52)
Number of x-ray visits	0.47 (0.95)^M^	0.08 (0.33)	0.39 (0.87)^M^	0.12 (0.44)	0.66 (1.15)^XL^	0.07 (0.32)
Number of different specialists visited (excluding lab and x-ray visits)*	2.45 (1.25)^XL^	1.20 (0.90)	2.47 (1.23)^L^	1.28 (0.97)	3.06 (1.34)^XL^	1.12 (0.86)
Number of days received a prescription medication	85.14 (107.15)^XL^	12.06 (25.27)	59.34 (97.68)^M^	20.48 (48.37)	112.95 (124.72)^XL^	11.52 (24.59)
Number of prescriptions	5.57 (8.50)^L^	0.96 (1.58)	4.25 (8.88)^M^	1.45 (2.97)	7.92 (12.34)^XL^	0.92 (1.54)
Days hospitalized (including children not hospitalized)*	0.34 (3.27)^S^	0.01 (0.25)	0.42 (3.75)^S^	0.02 (0.55)	0.88 (5.58)^M^	0.01 (0.14)

Table 3 describes maternal health outcomes across our child health groupings. Mothers of children with any of the three indicators showed consistently poorer health outcomes than mothers of children without these indicators. For the High Service Use grouping, effect sizes were usually trivial or small, with the exception of medium effect sizes for the number of physician visits and number of different 3-level ATC prescriptions filled. For the Diagnosis grouping, effect sizes were mostly trivial, with the exception of small effect sizes for number of physician visits, number of different specialists visited, and number of different 3-level ATC prescriptions filled. For the Both grouping, differences were generally larger with effect sizes similar to the High Service Use grouping, i.e. medium effect sizes for number of physician visits and number of different 3-level ATC prescriptions filled. The groups showed a similar pattern across chronic condition outcomes, with the High Service Use and Both groupings (but not the Diagnosis grouping) showing small effect sizes for presence of mood or anxiety disorder and any chronic condition.

**Table 3: Maternal health outcomes1:** ^1^ All numbers include non-users (e.g., days hospitalized includes those mothers with no hospitalizations, number of prescriptions includes those mothers with no filled prescriptions) ^S^ small effect size compared to corresponding “no” or “neither” groups ^M^ medium effect size compared to corresponding “no” or “neither” groups ^2^ Definition of mood or anxiety disorder from Brownell et al. 2012 [17]

	Child High Service Use		Child Diagnosis		High Service Use and Diagnosis combined	

	Yes	No	Yes	No	Both	Neither

	n = 29,480	n = 133,507	n = 19,892	n = 132,955	n = 8,665	n = 122,280
	(18%)	(82%)	(12%)	(88%)	(5%)	(75%)
**Physician visits (mean (sd))**						
Number of physician visits (excluding visits for birth/pregnancy and lab/x-ray)	11.04 (11.29)^M^	6.25 (8.14)	9.12 (10.17)^S^	6.83 (8.77)	11.24 (11.52)^M^	6.14 (8.08)
Number of lab visits	2.05 (2.79)^S^	1.33 (2.24)	1.79 (2.54)	1.41 (2.34)	2.07 (2.78)^S^	1.30 (2.24)
Number of x-ray visits	0.84 (1.26)^S^	0.53 (0.98)	0.73 (1.16)	0.57 (1.03)	0.87 (1.26)^S^	0.52 (0.98)
Number of different specialists visited (excluding for pregnancy lab/x-ray)	2.01 (1.36)^S^	1.51 (1.18)	1.87 (1.30)^S^	1.56 (1.21)	2.05 (1.40)^S^	1.49 (1.17)
**Hospitalization, excluding birth (mean (sd) or %))**						
% hospitalized	3.76	2.71	3.41	2.83	3.89^S^	2.68
Days hospitalized, excluding birth	0.25 (3.17)	0.16 (2.40)	0.19 (2.25)	0.17 (2.59)	0.25 (2.85)	0.16 (2.46)
**Prescription medication use (mean (sd) or %))**						
Number of days of prescription medications filled (excluding birth control)	107.46 (118.69)^S^	70.66 (105.37)	93.99 (115.37)	74.97 (107.67)	110.04 (120.27)^S^	69.66 (104.90)
Number of prescriptions (excluding birth control)	9.89 (34.41)	5.72 (24.39)	7.97 (24.79)	6.26 (26.75)	9.65 (22.67)	5.63 (24.21)
Number of different 3 level ATC’s	3.50 (3.40)^M^	2.12 (2.56)	2.92 (3.09)^S^	2.29 (2.73)	3.51 (3.45)^M^	2.09 (2.55)
% received medication for pain	21.47^S^	14.83	18.71	15.65	21.20^S^	14.65
% received medication for insomnia	4.57^S^	2.97	4.03	3.15	5.00^S^	2.94
**Chronic conditions (ICD-9) (mean (sd))**						
Mood or anxiety disorder^2^	21.87^S^	13.79	18.81	14.75	22.60^S^	13.59
% with any chronic condition	42.07^S^	27.90	37.13	29.52	42.38^S^	27.42

Table 4 examines associations between child health groupings and maternal health and service use outcomes, after controlling for child age, sex, maternal age, and proxies for family socio-economic status (living in lowest-income quintile area and receiving premium subsidy). We conducted regressions examining associations with four maternal outcomes: number of maternal physician visits (using linear regression), presence/absence of a maternal chronic condition, presence/absence of maternal mood or anxiety disorder, and maternal hospitalization (all three using logistic regression). After controlling for maternal and child demographics, results were consistent across all outcomes. Individually, each of the child health indicators was significantly associated with the maternal outcomes, while the combination represented by the Both grouping accounted for greater variance than either individually (full stepwise models in appendix).

**Table 4: Beta estimates (standard errors) and odds ratios (95% confidence intervals) from regression analyses predicting maternal outcomes:** ^*^ p<0.05 ^1^ Estimates are beta coefficients (standard errors). ^2^ Estimates are odds ratios (confidence intervals). Note: R-Square values for the binary outcomes (i.e., presence/absence of a chronic condition, presence/absence of mood or anxiety disorder, and maternal hospitalization) are pseudo r-squares based on Nagelkerke (1991)[33] adjusted coefficient.

		Number of physician visits (excluding birth/pregnancy and lab/x-ray visits)^1^	Any chronic condition (No/Yes)^2^	Mood or anxiety disorder (No/Yes)^2^	Hospitalization (No/Yes)^2^

Variable					
Age of mother (years)		0.00 (0.00)	1.02 (1.02-1.03)*	0.99 (0.99-0.99)*	0.99 (0.98-0.99)*
Family lives in lowest-income quintile area		0.39 (0.05)*	1.02 (0.99-1.05)	0.94 (0.91-0.97)*	1.04 (0.97-1.12)
Family receives premium subsidy		3.64 (0.05)*	1.67 (1.63-1.71)*	1.61 (1.56-1.66)*	1.81 (1.70-1.92)*
Age of child (years)		0.04 (0.02)*	1.01 (1.00-1.01)	1.01 (1.00-1.02)*	1.03 (1.01-1.05)*
Child is female		0.19 (0.04)*	1.04 (1.02-1.07)*	1.01 (0.98-1.03)	0.96 (0.91-1.02)
Child health category					
	Neither indicator	Ref.	Ref.	Ref.	Ref.
	Diagnosis indicator only	1.33 (0.09)*	1.30 (1.25-1.36)*	1.20 (1.14-1.27)*	1.14 (1.02-1.27)*
	High Service Use only	4.57 (0.07)*	1.87 (1.81-1.93)*	1.69 (1.63-1.76)*	1.33 (1.23-1.44)*
	Both indicators	4.87 (0.10)*	1.90 (1.82-1.99)*	1.81 (1.71-1.91)*	1.41 (1.26-1.58)*
R-Square		7.56%	3.84%	2.37%	1.33%

## Discussion

In this study, we sought to establish the feasibility of using administrative health data to explore differences in health and service use of caregivers of children with and without health problems. We explored whether two child health indicators, previously shown to differentiate children on important child health outcomes [16, 19], could be used together to create meaningful child health groupings and also whether these groupings were associated with differences in the health of mothers.

Both indicators can claim to group children in a non-condition specific manner. Our results showed that using a child grouping of those with or without a Diagnosis of a major and/or chronic condition showed the weakest associations with maternal outcomes, with consistent but small effects across the range of maternal health outcomes examined in univariate analyses. After controlling for child and maternal demographics, this indicator maintained a small association with number of maternal physician visits, presence of maternal major and/or chronic ADGs, presence/absence of maternal mood or anxiety disorder, and maternal hospitalization. This finding suggests that child diagnosis alone might not be the strongest available indicator for exploring associations with maternal health.

Grouping children based on High Service Use showed somewhat larger effects in both univariate and regression analyses for the four maternal outcomes. Children who were in the Both grouping (i.e. high service use and a diagnosis) showed the strongest association with all four maternal outcomes in multivariate regressions, but effect sizes were similar for children in the high service use only group. Overall, variance in maternal outcomes explained by the models was quite low (1.3 – 7.6%). While our analytical goal was comparison of the indicators rather than maximizing variance explained, the low overall variance explained suggests that other factors including physical and social determinants may play an additional important role in explaining maternal health.

Our estimates of the prevalence of various maternal chronic conditions are in the range of estimates from other studies using survey and administrative data. For example, our rates of diabetes, heart disease, and mood or anxiety disorder are similar to the estimates found using health surveillance data [34-36]. However, some survey-based estimates are higher than those found in our study. For example, using the Canadian Community Health Survey, 8.6% of 35 to 44-year-old women reported having a diagnosis of arthritis, compared to 0.63 to 1.23% in our study. Similarly, about 9% of 35 to 44-year olds reported an asthma diagnosis, compared to 3.09% to 5.75% in our study. Survey estimates may be higher because of a broader time frame for having received a diagnosis (“ever been diagnosed” compared to a single year in the present study). Our estimates of number of maternal physician visits were somewhat higher than other estimates based on outpatient samples.[37] However, our data included inpatient visits, which would be high among some mothers having children with above average medical needs. As well, differences in sample frame, data collection method, and reporting bias can all contribute to the different estimates from surveys as compared to administrative data. Further validation work is needed inform the extent to which administrative data can yield externally valid prevalence information, and under what circumstances. At present, careful interpretation of these results should focus primarily on differences between child groupings.

### Limitations

A number of limitations of this work warrant consideration. Our administrative health data are known to suffer from errors such as incomplete coding, underrepresentation of people with poor access to the health system or who are not represented by MSP billing information (i.e. refugees, new immigrants, people living in rural and remote areas, Indigenous people), and variability in the validity of data of different clinical conditions [38]. Validity of administrative data can vary, with less known about validity of pediatric data; [39, 40], this can be a particular challenge in our work that involves both child and adult data. Further work is required to explore whether these factors may have contributed to some of the differences in rates of maternal health conditions we observed in administrative data compared to survey data.

This work shows that associations between child and maternal health outcomes can be explored with administrative data, and furthers our understanding of the interconnected health of children and their mothers. Understanding these relationships more clearly will enable us to provide better and more timely support and services to mothers and families challenged by children with health problems. To date, however, we cannot make strong claims about the causal pathways underlying these associations. For example, associations identified between our child High Service Use indicator and maternal service use outcomes (e.g. maternal physician visits) may stem from some individual providers that treat both the child and the mother tending to engage more services overall, or mothers seeking out more services both for themselves and their children. Associations may stem from maternal health problems (e.g. through genetic or other family health predispositions), rather than the caregiving situation per se, or from the health of the mother affecting the health of the child. In part, the nature of the data precludes stronger conclusions; our data examine only a 1-year cross-section of information. Examining health administrative data longitudinally, perhaps exploring maternal health prior to and after the birth of children with or without health problems, may allow for a better exploration of the extent to which caring for a child with health problems is linked causally to maternal health. Our group is exploring this approach [21].

Classifying childhood health problems non-categorically rather than by individual diagnoses assumes that different child health conditions can have common implications for both child and caregiver health. The approach has been shown to be useful in the context of survey data, but in the context of administrative health data is less well understood. First, it is complicated by the fact that administrative data are not uniform in quality or completeness [38]. In Canada, services for mental health conditions are not well captured in administrative data since only the most severe conditions are covered by provincial health care plans, while many other services are paid for out-of-pocket and are not captured by BC administrative health data. This may partly explain weak associations with mental health outcomes found in the present study. Second, we have shown previously[19] that administrative data often do not clearly address important issues such as condition severity and function, essential elements of non-categorical descriptions of health. Understanding the potential biases implied by these issues, developing new tools for categorizing severity and function, and better understanding when and how administrative health data can reliably provide non-categorical indicators, are important areas for further study.

## Conclusion

This work employs administrative health data to develop new non-categorical measures of child health (for others see [16, 41-46]), and to our knowledge is the first to use such measures to study the health of family members of children with health problems. The work demonstrates the feasibility of the approach and highlights some of the challenges in using such data to compare child health groupings involving ranges of clinical conditions, but which nevertheless share caregiving challenges. Perhaps most importantly, it demonstrates the feasibility of using administrative health data as a research tool that can target family health issues. Our results use new methods to replicate findings that mothers of children who are both high service users and have major and/or chronic conditions may be at greatest risk for health issues themselves. More broadly, the work suggests service providers may wish to consider the impact of the child’s health on caregiver health, and leads to consideration of the possible benefits of a comprehensive service provision model that addresses both child and family health service needs together.

## Ethics approval

This work was reviewed by the Ottawa Health Science Network Research Ethics Board (OHSN REB) (approval number:2009738-01H)

## Authors’ contributions

J. C. Brehaut originated and designed the study, interpreted the data, drafted the article, supervised the study, and supplied administrative, technical, and material support. A. Guèvremont drafted parts of the article, analyzed and helped interpret the data; and provided statistical expertise. All other authors provided specific content or methodological expertise throughout the project, including interpretation of the findings and provision of critical revisions of article.

## Supplementary Appendices

Supplementary Table 1. Description of child and maternal health outcomes measured

Supplementary Table 2. Full regression results. Estimates associated with maternal outcomes

## Abbreviations

Medical Services Plan (MSP)

Discharge Abstract Database (DAD)

Children with Special Health Care Needs (CSHCN)

Aggregated Diagnosis Groups (ADG)

Drug Identification Number (DIN)

Anatomical Therapeutic Chemical (ATC) Classification Code

International Classification of Diseases v. 9 (ICD-9)
