# Supplementary material for: Using Canadian administrative health data to examine the health of caregivers of children with and without health problems: A demonstration of feasibility
Source: Int J Popul Data Sci. 2019 Apr 2;4(1):584. doi: 10.23889/ijpds.v4i1.584 (PMC7479927; doi:10.23889/ijpds.v4i1.584)
Supplement: Supplementary Table 1. Description of child and maternal health outcomes measured [file ijpds-04-584-s001.pdf]

Appendix 1: Description of child and maternal health outcomes measured

**Physician visits (based on MSP data)**		**Child**	**Mother**
Number of physician visits	Number of physician visits in 2006. A physician visit is based on number of unique records by date and specialty, excluding visits for pregnancy. Pregnancy or birth related visits excluded any visit coded with the following ICD-9 or Popdata BC codes: 630 to 679, 760 to 779, V20 to V28, Popdata BC codes 30B, 31B, 32B, 33B, 34B, 35B, 36B, 37B, 38B, 34A, 23B, 08A, 12B, 15B, 16B, 10B, 18B, 11B, 19B.	✓	✓
Number of lab visits (mean)	Number of lab visits in 2006, based on the number of visits with a code of “L01”.	✓	✓
Number of x-ray visits (mean)	Number of lab visits in 2006, based on the number of visits with a code of “X01”.	✓	✓
Number of different types of physicians visited	Number of different types of physicians visited, including general practitioners, in 2006. Pregnancy or birth related visits excluded any visit coded with the following ICD-9 or Popdata BC codes: 630 to 679, 760 to 779, V20 to V28, Popdata BC codes 30B, 31B, 32B, 33B, 34B, 35B, 36B, 37B, 38B, 34A, 23B, 08A, 12B, 15B, 16B, 10B, 18B, 11B, 19B.	✓	✓
**Hospitalizations (based on hospital data)**			
Hospitalized (%)	Hospitalized overnight in 2006, excluding hospitalization for birth or pregnancy as indicted by the following ICD-9 or Popdata BC codes: 630 to 679, 760 to 779, V20 to V28, Popdata BC codes 30B, 31B, 32B, 33B, 34B, 35B, 36B, 37B, 38B, 34A, 23B, 08A, 12B, 15B, 16B, 10B, 18B, 11B, 19B		✓
Days hospitalized	Number of nights hospitalized in 2006, excluding for pregnancy or birth, including those who were not hospitalized	✓	✓

**Prescription medication use (based on PharmaNet data)**			
Number of days used a prescription medication (excluding birth control)	A day of medication use was defined based on the date the prescription was filled and the number of days of medication supplied. One day of prescription medication use could include one or more prescriptions subscribed for that day.	✓	✓
Received medication for pain	Received at least one prescription for a medication with Anatomical Therapeutic Classification (ATC) System code “N02”		✓
Received medication for insomnia	Received at least one prescription for a medication with ATC code “N05C”		✓
Number of different level 3 ATC codes (excluding birth control)	ATC codes divide active substances into groups according to the organ or system on which they act and their therapeutic, pharmacological and chemical properties. Level-3 ATC codes represent major therapeutic or pharmacological subgroups.		✓

Number of prescriptions filled (excluding birth control)	Number of prescriptions filled in 2006.	✓	✓
**Maternal chronic conditions based on ICD-9 codes (based on MSP data, except for “mood or anxiety disorder”)**			
Mood or anxiety disorder[a] (%)	As in Brownell et al. (2012, 2015)[17, 47], Smith et al. (2013)[48], and Chartier et al. (2016)[49], a mother was considered to have a mood and anxiety disorder if she met at least one of the following criteria in 2006:<br>• one or more hospitalizations for depressive disorder, affective psychoses, neurotic depression, or adjustment reaction (ICD-9 codes 296.1-296.8, 300.4, 309, 311) or with a diagnosis for an anxiety state, phobic disorder, or obsessive-compulsive disorder (ICD-9 codes 300.0, 300.2, 300.3, 300.7);<br>• one or more hospitalizations with a diagnosis for anxiety disorders (ICD-9 code 300) AND one or more prescriptions for an antidepressant or mood stabilizer including medications with the ATC codes N05AN01, N05BA, N06A.<br>• one or more physician visits with a diagnosis for depressive disorder or affective psychoses: ICD-9 codes 296, 311;<br>• one or more physician visits with a diagnosis for anxiety disorders: ICD-9-CM code 300 AND one or more prescriptions for an antidepressant or mood stabilizer, including medications with the ATC codes N05AN01, N05BA, N06A.		✓
Any chronic condition (%)	One or more of the following chronic conditions based on ICD-9 codes (listed in parentheses): allergy (287, 477, 995.27 or 995.3), arthritis (714), asthma (493), back problem (724), bronchitis (490, 491, 492, 466.0, 506.0), cancer (140-208,235-239), diabetes (249, 250), hearing problem (388, 389), heart disease (393-398, 401-405,410-414, 415-417,420-429), herpes (054, 771.2), hypertension (401, 796.2), injury (E800.0-E999), migraine (346), sinusitis (473), ulcer (531, 532), vision problem (365, 368).		✓

[a] Although previous studies have demonstrated face validity as well as predictive validity, this definition has not been compared to a “gold standard” such as a chart review or a clinical database that includes patients who have been indicated as having the disorder.
