# Supplementary material for: Using Canadian administrative health data to examine the health of caregivers of children with and without health problems: A demonstration of feasibility
Source: Int J Popul Data Sci. 2019 Apr 2;4(1):584. doi: 10.23889/ijpds.v4i1.584 (PMC7479927; doi:10.23889/ijpds.v4i1.584)
Supplement: Supplementary Table 2. Full regression results. Estimates associated with maternal outcomes [file ijpds-04-584-s002.pdf]

Appendix 2: Full regression results. Estimates and overall R^2's associated with maternal outcomes

Predicting mother's number of physician visits

Variable	**Mother's Age + SES**	**Mother's Age, Child Age, Sex, + SES**	**Demographics + Diagnosis Alone**	**Demographics + Services Alone**	**Demographics, Diagnosis and Services**
Age of mother	0.00 (0.00)	0.00 (0.00)	0.00 (0.00)	0.00 (0.00)	0.00 (0.00)
Family lives in lowest-income quintile area	0.40 (0.06)*	0.40 (0.06)*	0.41 (0.06)*	0.39 (0.05)*	0.39 (0.05)*
Family receives premium subsidy	3.90 (0.05)*	3.90 (0.05)*	3.87 (0.05)*	3.64 (0.05)*	3.64 (0.05)*
Age of child		0.01 (0.02)	0.02 (0.02)	0.04 (0.02)*	0.04 (0.02)*
Child is female		0.05 (0.04)	0.08 (0.04)	0.18 (0.04)*	0.19 (0.04)*
Diagnosis indicator (vs. not)			2.20 (0.07)*		
High Service Use (vs. not)				4.54 (0.06)*	
Child health category					
Neither indicator					ref.
Diagnosis indicator only					1.33 (0.09)*
High service use only					4.57 (0.07)*
Both indicators					4.87 (0.10)*
R-square	3.66%	3.66%	4.31%	7.42%	7.56%

*p<0.05

Predicting any chronic condition

Variable	Mother's Age + SES	+ Child Age, Sex	Demographics + Diagnosis Alone	Demographics + Services Alone	Demographics, Diagnosis and Services
Age of mother	1.02 (1.02-1.03)*	1.02 (1.02-1.03)*	1.02 (1.02-1.03)*	1.02 (1.02-1.03)*	1.02 (1.02-1.03)*
Family lives in lowest-income quintile area	1.02 (0.99-1.05)	1.02 (0.99-1.05)	1.02 (0.99-1.05)	1.02 (0.99-1.05)	1.02 (0.99-1.05)
Family receives premium subsidy	1.72 (1.68-1.76)*	1.72 (1.68-1.76)*	1.71 (1.67-1.75)*	1.67 (1.63-1.71)*	1.67 (1.63-1.71)*
Age of child		1.00 (0.99-1.01)	1.00 (1.00-1.01)	1.01 (1.00-1.01)	1.01 (1.00-1.01)
Child is female		1.02 (1.00-1.04)*	1.03 (1.01-1.05)*	1.04 (1.02-1.06)*	1.04 (1.02-1.07)*
Diagnosis indicator (vs. not)			1.40 (1.35-1.44)*		
High Service Use indicator (vs. not)				1.84 (1.79-1.88)*	
Child health category					
Neither indicator					ref.
Diagnosis indicator only					1.30 (1.25-1.36)*
High Service Use indicator only					1.87 (1.81-1.93)*
Both indicators					1.90 (1.82-1.99)*
Pseudo r-square	2.01%	2.01%	2.38%	3.71%	3.84%

*p<0.05

Predicting mood or anxiety disorder

Variable		Mother's Age + SES	+ Child Age, Sex	Demographics + Diagnosis Alone	Demographics + Services Alone	Demographics, Diagnosis and Services
Age of mother		0.99 (0.99-0.99)*	0.99 (0.99-0.99)*	0.99 (0.99-0.99)*	0.99 (0.99-0.99)*	0.99 (0.99-0.99)*
Family lives in lowest-income quintile area		0.94 (0.91-0.97)*	0.94 (0.91-0.97)*	0.94 (0.91-0.97)*	0.94 (0.90-0.97)*	0.94 (0.91-0.97)*
Family receives premium subsidy		1.66 (1.61-1.71)*	1.66 (1.61-1.71)*	1.65 (1.60-1.70)*	1.61 (1.56-1.66)*	1.61 (1.56-1.66)*
Age of child			1.01 (1.00-1.02)	1.01 (1.00-1.02)	1.01 (1.00-1.02)*	1.01 (1.00-1.02)*
Child is female			0.99 (0.96-1.01)	0.99 (0.96-1.02)	1.00 (0.98-1.03)	1.01 (0.98-1.03)
Diagnosis indicator (vs. not)				1.33 (1.28-1.38)*		
High Service Use indicator (vs. not)					1.70 (1.65-1.75)*	
Child health category						
	Neither indicator					ref.
	Diagnosis indicator only					1.20 (1.14-1.27)*
	High Service Use indicator only					1.69 (1.63-1.76)*
	Both indicators					1.81 (1.72-1.91)*
Pseudo r-square		1.26%	1.27%	1.48%	2.32%	2.38%

*p<0.05

Predicting hospitalization

Variable	Mother's Age + SES	+ Child Age, Sex	Demographics + Diagnosis Alone	Demographics + Services Alone	Demographics, Diagnosis and Services
Age of mother	0.99 (0.99-1.00)*	0.99 (0.98-0.99)*	0.99 (0.98-0.99)*	0.99 (0.98-0.99)*	0.99 (0.98-0.99)*
Family lives in lowest-income quintile area	1.05 (0.97-1.12)	1.04 (0.97-1.12)	1.04 (0.97-1.12)	1.04 (0.97-1.12)	1.04 (0.97-1.12)
Family receives premium subsidy	1.87 (1.76-1.99)*	1.86 (1.75-1.98)*	1.86 (1.75-1.98)*	1.83 (1.72-1.95)*	1.83 (1.72-1.95)*
Age of child		1.02 (1.00-1.05)*	1.03 (1.00-1.05)*	1.03 (1.01-1.05)*	1.03 (1.01-1.05)*
Child is female		0.95 (0.90-1.01)	0.96 (0.90-1.01)	0.96 (0.91-1.02)	0.96 (0.91-1.02)
Diagnosis indicator (vs. not)			1.20 (1.10-1.30)*		
High Service Use indicator (vs. not)				1.34 (1.25-1.43)*	
Child health category					
Neither indicator					ref.
Diagnosis indicator only					1.14 (1.01-1.27)*
High Service Use indicator only					1.33 (1.23-1.44)*
Both indicators					1.41 (1.26-1.58)*
Pseudo r-square	1.17%	1.19%	1.23%	1.36%	1.37%

*p<0.05
